# The effects of microclimatic winter conditions in urban areas on the risk of establishment for *Aedes albopictus*

**DOI:** 10.1038/s41598-022-20436-9

**Published:** 2022-09-24

**Authors:** Damiana Ravasi, Francesca Mangili, David Huber, Massimiliano Cannata, Daniele Strigaro, Eleonora Flacio

**Affiliations:** 1 Department for Environment Constructions and Design, Vector Ecology Unit, Institute of Microbiology (IM), University of Applied Sciences and Arts of Southern Switzerland (SUPSI), 6850 Mendrisio, Switzerland; 2 Department of Innovative Technologies, Dalle Molle Institute for Artificial Intelligence Studies (IDSIA), University of Applied Sciences and Arts of Southern Switzerland (SUPSI), 6962 Lugano-Viganello, Switzerland; 3 Department for Environment Constructions and Design, Institute of Earth Sciences (IST), University of Applied Sciences and Arts of Southern Switzerland (SUPSI), 6850 Mendrisio, Switzerland

**Keywords:** Entomology, Machine learning, Urban ecology, Invasive species, Ecological modelling, Climate-change ecology

## Abstract

The tiger mosquito, *Aedes albopictus*, has adjusted well to urban environments by adopting artificial water containers as oviposition sites. Its spread in temperate regions is favoured by the deposition of cold-tolerant diapausing eggs that survive winter temperatures to a certain degree. The probability of establishment in new geographical areas is estimated using predictive models usually based on meteorological data measured at coarse resolution. Here, we investigated if we could obtain more precise and realistic risk scenarios for the spread of *Ae. albopictus* when considering the winter microclimatic conditions of catch basins, one of the major sites of oviposition and egg overwintering in temperate urban areas. We monitored winter microclimatic conditions of catch basins in four Swiss cities and developed a regression model to predict the average microclimatic temperatures of catch basins, based on available meteorological parameters, accounting for the observed differences between cities. We then used the microclimatic model to correct the predictions of our previously developed risk model for the prediction of *Ae. albopictus* establishment. Comparison of the predictive model’s results based on local climate data and microclimate data indicated that the risk of establishment for *Ae. albopictus* in temperate urban areas increases when microhabitat temperatures are considered.

## Introduction

In the last three decades, the tiger mosquito, *Aedes albopictus* (Skuse), originally found in natural habitats of Southeast Asia, has spread to all continents except Antarctica through increased human global trade, travel and rising temperatures associated with climate change. Thanks to its ecological plasticity, the species has adjusted well to urban environments by adopting artificial water containers, such as watering cans, plastic drums and catch basins of stormwater drains, as breeding sites^1,2^. In addition, its capability to develop cold-tolerant diapausing eggs able to survive winter temperatures has allowed its establishment in temperate regions^3^. Its global expansion, combined with its competence for a range of tropical viral and parasitic agents (e.g., dengue and chikungunya viruses), makes it a major global health concern^4^. Efficient local monitoring and control are therefore essential to contain its spread and establishment.

Mosquitoes are ectothermic organisms. Consequently, meteorological factors, such as temperature, precipitations, air humidity, solar radiation, soil moisture and wind speed, affect their development processes, activity, survival and altogether their population dynamics^5–9^. Ecological (or environmental) niche models are used to relate known invasive mosquito’s occurrence data with meteorological factors and other environmental information to predict their distributional potential in geographic areas not yet occupied^10–13^. The risk maps produced through these models can support strategic management decisions, such as the surveillance and control interventions in areas at higher risk of invasion.

Most of the current predictive models are based on climatic data originating from weather stations or remotely sensed land surface temperature data. However, the spatial and temporal resolutions of these datasets might be too coarse to capture the actual microclimatic conditions experienced by the organisms within their habitats. This may lead to inaccuracy in estimating the potential range of distribution of a species^14–16^.

For instance, the range expansion of *Ae. albopictus* in temperate regions is highly dependent on the survival of diapausing eggs to winter cold temperatures^17^. However, one of the main oviposition sites for *Ae. albopictus*, namely the catch basin of urban stormwater drains^18,19^, could present microclimatic conditions which are decoupled from the local climate measured by weather stations.

In a previous work, we investigated whether catch basins in Swiss cities north of the Alps, where *Ae. albopictus* was not established at the time, could potentially offer better temperature conditions for the overwintering of eggs, compared to external weather conditions that have been used to model suitability maps. The results showed that winter temperatures in catch basins were higher and more stable than external temperatures measured at nearby weather stations^20^. In addition, winter temperatures were higher in urban catch basins north of the Alps compared to catch basins south of the Alps, in which *Ae. albopictus* eggs do overwinter, highlighting the potential importance of taking into account these microhabitat conditions in predictive distribution models^20^. A small number of studies^21–23^ used a combination of local climate data, geospatial data of land use and networks of microclimate sensors to estimate the climatic conditions of microhabitats. Traditionally, these microclimate models are used to feed temperature-dependent mechanistic models derived from mosquitoes’ thermal performance curves with good estimates of the temperatures met in their microhabitats, which are, instead, very poorly approximated by external, low-resolution interpolations of weather station data. However, in the present work, the risk model is not based on mechanistic models based on local temperatures, but on a data-driven model learned on local gridded temperature data. In this context, the relation between microclimate data and low-resolution temperatures which more directly affect mosquito’s establishment is implicitly accounted for by the model and therefore should not need to be explicitly estimated by microclimatic temperature models. However, the relation between local and micro climatic conditions can vary from the region where the model is developed to those where it is applied, and therefore, the climatic conditions of microhabitats should be considered and analyzed also when data-driven models for mosquitoes’ establishment are used. At present, to our knowledge, studies that take into account urban microclimatic conditions in data-driven ecological niche modelling are still lacking, the main reason for this perhaps being the general unavailability of microclimatic data and the difficulty to collect this type of data in terms of costs and labour^16^.

In Switzerland, *Ae. albopictus* appeared in the Canton of Ticino (hereafter referred to as Ticino), in the southern side of the Alps, in 2003 and is currently well established in most urban areas of the canton^24–26^. North of the Alps, possible foci of introduction have been recently observed in different cantons and the invasion of urban areas in the next years is likely. Small populations of the mosquito have already become firmly established since 2018 in two areas in the city of Basel^27^. *Ae. albopictus* has also been sighted in the cities of Zurich, Geneva and Bern (Swiss Mosquito Network, http://www.mosquitoes-switzerland.ch (accessed on 19 October 2021)). To help local authorities optimizing surveillance and control, we recently developed a predictive risk model to evaluate the probability of establishment in Switzerland and more finely in the cities of Basel and Zurich^28^. In the training process, the model, a Lasso-regularized logistic regression ensemble, used the long-term dataset of *Ae. albopictus* presence–absence records in Ticino as response variable. Seventy-nine socioenvironmental factors potentially relevant to the distribution of *Ae. albopictus* were evaluated by the model as candidate predictors. These included static attributes for cell terrain morphology, land use coverage, and permanent host population, as well as dynamic features such as meteorological variables and travel distance from the nearest cell with establishment. The temperature predictors used in the model were derived from the MeteoSwiss spatial climate daily datasets (source: MeteoSwiss) and had a spatial resolution of 1 km. Among the ten explanatory features identified as most informative for the prediction of establishment of *Ae. albopictus* in Ticino, two were related to cold-season temperatures. This result highlighted the need of considering the cold-season microclimatic conditions in diapausing sites when predicting the risk of the establishment.

Therefore, to obtain more precise and realistic risk scenarios for the spread of *Ae. albopictus* in the Swiss cities Basel, Lausanne and Zurich, we integrated microclimatic data into the previously developed ecological niche model with the goal of understanding if and how the predictions changed when the winter microclimatic temperatures of catch basins were considered. As it is unrealistic to constantly monitor all the microhabitats of these cities, we have developed an approach aimed at introducing a correction in the risk predictions based on the average microclimatic conditions of the catch basins inside the cities of interest. In a first step, we developed and implemented a Wireless Sensor Network (WSN) based on Internet of Things (IoT) technologies to monitor the microclimatic conditions of stormwater catch basins. The successful development and validation of the WSN, described in a separate publication^29^, allowed us to: (1) model the relation between meteorological temperatures and microclimatic temperatures in each monitored catch basin and compare these models across the selected Swiss cities; (2) develop a regression model to predict the average microclimatic temperatures of catch basins, based on available meteorological parameters, accounting for the observed differences between cities; (3) use the microclimatic model to correct the predictions of the previously developed risk model for the prediction of *Ae. albopictus* establishment.

## Results

### Microclimatic vs. meteorological temperature

In total, we obtained 82,008 temperature measurements from 39 sensor devices (29 installed in catch basins and ten installed in external habitats) over a period of 91 days, from the beginning of December 2019 to the end of February 2020 (Supplementary Table S2). Data from eleven catch basins and five external habitats could not be used due to loss or failure of the sensor devices.

**Figure 1:**
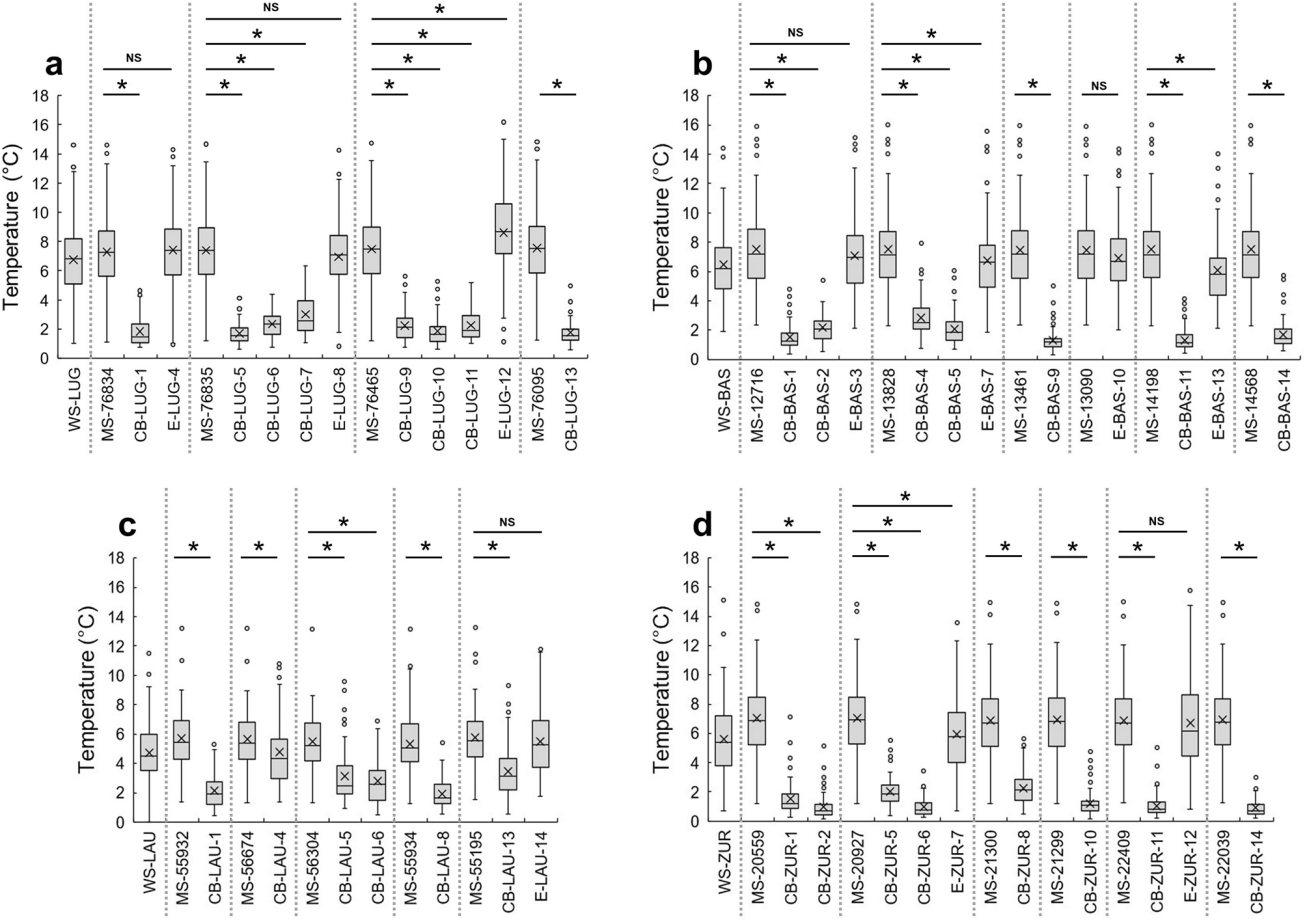
Daily temperature ranges in the cities of Lugano (**a**), Basel (**b**), Lausanne (**c**) and Zurich (**d**). The x marks within the boxes represent the means. Temperatures were recorded at permanent weather stations (WS), MeteoSwiss cells (MS) and corresponding catch basins (CB) and external potential warm-season resting habitats (E) (Supplementary Table S1). *: Significant differences. NS: differences not significant.

We compared the daily temperature data of catch basins and external habitats to the corresponding cells of the gridded MeteoSwiss (MS) temperature data. We observed a constantly smaller daily temperature fluctuation in catch basins, compared to the gridded temperatures (Fig. 1).

In the catch basins in Basel, Lugano and Zurich, the amount of daily temperature variation was comprised on average between 0.9 and 3.0 °C. In the catch basins in Lausanne, this variation was slightly wider, ranging between 1.9 and 4.8 °C. In the gridded data, the variation was significantly larger, ranging from 5.3 to 7.5 °C. The reduced temperature variation inside catch basins, which is mostly due to increased minimum temperatures, creates a favourable microclimate for the survival of diapausing eggs. The average daily variation measured by permanent weather stations was similar to the gridded data and varied between 4.7 and 6.7 °C. The average daily temperature range in the external habitats varied between 5.5 and 8.6 °C and did not differ significantly from the gridded data except in one case (LUG-12-E), where its median was significantly larger and in three cases (BAS-7-E, BAS-13-E and ZUR-7-E), where it was significantly smaller.

In all the cities, the median daily minimum temperature was significantly higher in catch basins compared to the gridded temperatures, confirming the warming effect of catch basins on cold temperature (Supplementary Fig. S3). The only exceptions were two catch basins (i.e., LUG-11-CB and LAU-13-CB), for which the difference was not significant, and one catch basin (i.e., LUG-7-CB), for which the median daily minimum temperature was significantly lower than the median outside temperature. In all locations, both inside and outside catch basins, daily minimum temperatures never dropped below – 4.7 °C.

The warming effect of catch basins was less present for average and maximum temperature. In Basel, Lausanne and Zurich, differences between the median daily average temperature of gridded data and catch basins were usually not significant, and, when significant, they showed significantly higher medians in catch basins except in one case in Lausanne (Supplementary Fig. S4). In Lugano, the catch basins’ daily mean temperature was significantly lower than the gridded temperature, except in two cases where the difference was not significant (Supplementary Fig. S4). This was probably due to the fact that the maximum daily temperatures obtained from gridded data in Lugano were much higher than the ones measured in the catch basins (data not shown). The median daily mean temperature in the external habitats did not differ significantly from the gridded temperature, except in one case in Lausanne.

**Figure 2:**
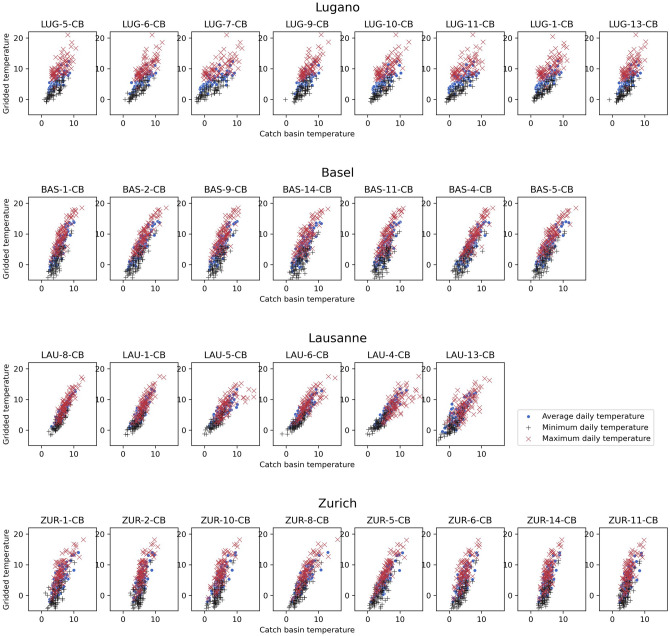
Scatterplots of the gridded temperatures versus catch basin temperatures.

The scatterplots in Fig. 2 show that, for all temperature data series (i.e., daily average, minimum and maximum temperatures), the relation between catch basins’ temperatures and gridded temperatures can be reasonably well approximated by a linear model.

Figure 2 also confirms that, while gridded temperatures can reach lower values in Basel and Zurich (with minima going below − 4 °C), compared to Lugano (which never reaches − 2 °C), the minimum values reached by the minimum, mean and maximum series inside the catch basins are not much different in the four cities.

**Figure 3:**
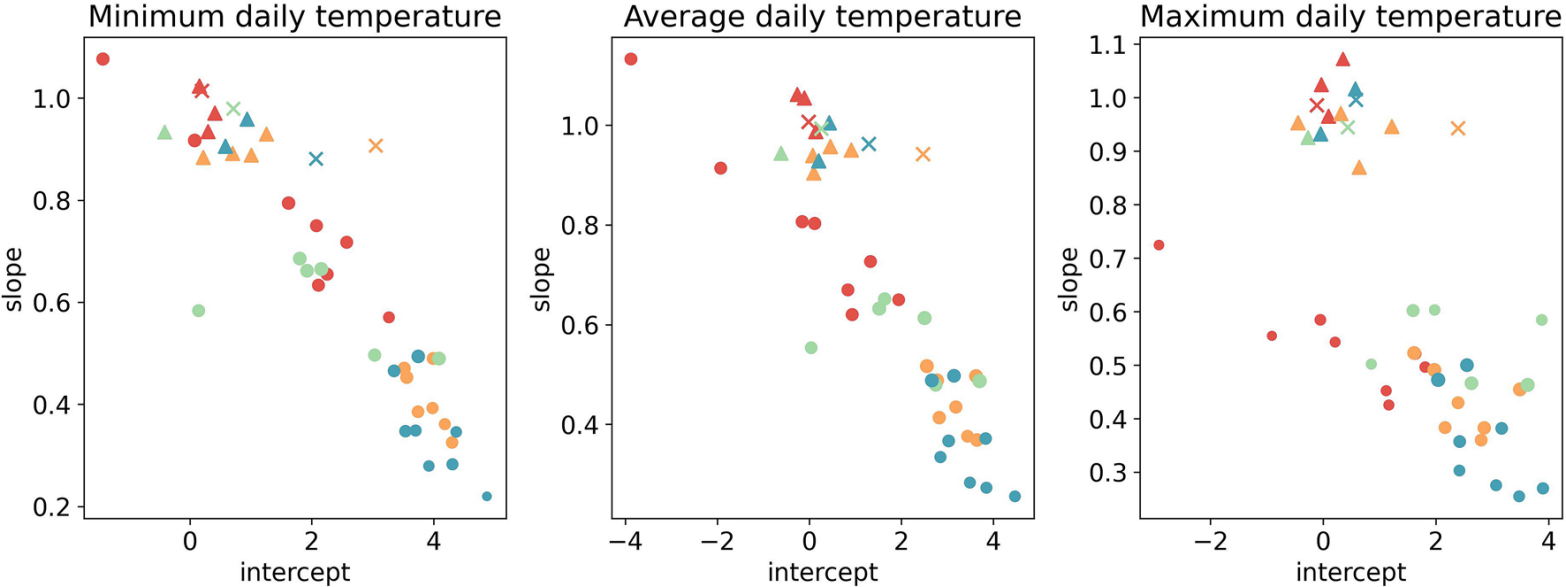
Scatterplots of the slopes ${m}_{i}$ versus the intercepts ${q}_{i}$ of the regression lines ${T}_{i}={m}_{i}{ T}_{MS}+{q}_{i}$ modelling the relation between gridded minimum (left), mean (centre) and maximum (right) daily temperatures and catch basins (dots), weather station (x) and external (triangle) temperatures for the cities of Lugano (blue), Basel (green), Lausanne(cyan) and Zurich (red).

The linear relationship observed between gridded temperatures and catch basin temperatures motivated the choice of fitting a linear regression model to the data from each catch basin. Similar models were built also for the relation between gridded temperatures and weather stations and external sensors temperature measurements. The slopes (y axis) and the intercepts (x axis) obtained are shown in Fig. 3.

As expected, slopes and intercepts of the weather station and external habitats models are always quite close to, respectively, 1 and 0, meaning that the external temperatures are very similar to the gridded temperatures. The parameters obtained for the catch basins have instead, in the large majority of cases, a slope smaller than 1 and an intercept higher than 0, showing that they tend to reduce the variations of external temperatures. This effect is more pronounced in Basel and Zurich, as we see from the fact that the coefficients obtained for these two cities cluster in the bottom right region of the scatterplots, i.e., around higher values of the intercept and smaller values of the slope. Instead, the regression models’ parameters of Lugano’s and Lausanne’s catch basins are located mostly in the upper left side, except for the maximum temperatures of Lausanne for which similar slopes as for Lugano but higher intercepts were obtained (Fig. 3). This highlighted a difference in the relation between catch basin’s microclimates and gridded temperatures in the different cities. We have verified with a MANOVA test that the difference between the mean slope and intercept of catch basins located in different cities is significant (*p*-value < 10^–4^) both for the minimum, mean and maximum daily temperatures. Similar results were obtained also when we took into consideration the variability in the illuminance arriving at the sensor device by including the illuminance predictor in the regression model (results not shown).

**Figure 4:**
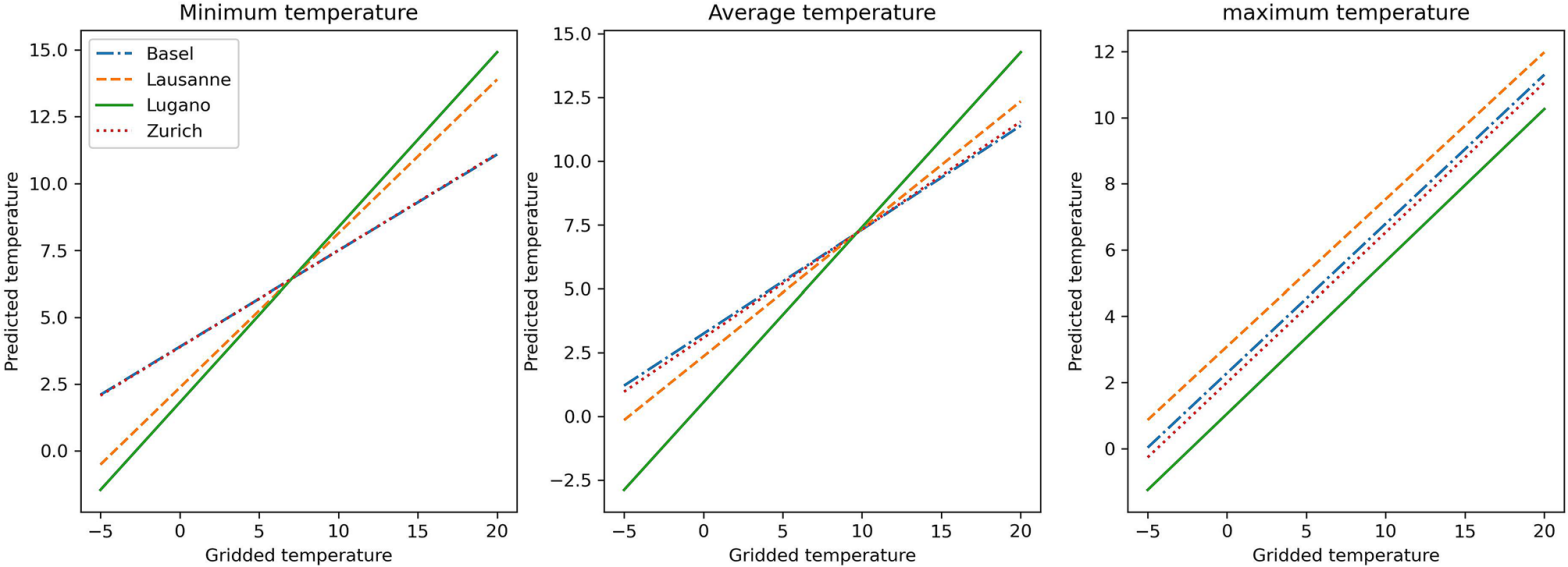
Regression lines obtained from the mixed effects model accounting for the city slope and intercept random effects.

Based on these differences between cities, we have trained a mixed effects model that transformed the gridded temperatures to mirror the average behaviours of the observed catch basins. The regression models of Eq. (1) obtained for the four cities for each temperature series are shown in Fig. 4. The normalized relative likelihood of this model compared to a linear mixed effects model including only the catch basin random effect is 0.995, confirming that there is a significant random effect associated to the city^30^.

From the regression lines in Fig. 4, one can see that both for the minimum and mean temperature series, the slope is smaller for Basel and Zurich, while the intercept is higher, confirming the strongest power to mitigate external temperature variations. As for the maximum temperature, there is no random effect for the slope, each city implying only a different offset in the catch basin temperature with respect to the external ones. In particular, such an offset is almost zero for Basel and Zurich, whereas it is negative for Lugano, showing a cooling effect of this city’s catch basins, and positive for Lausanne, where catch basins show instead a warming effect on the maximum temperatures.

### Outputs of the ecological niche model

**Figure 5:**
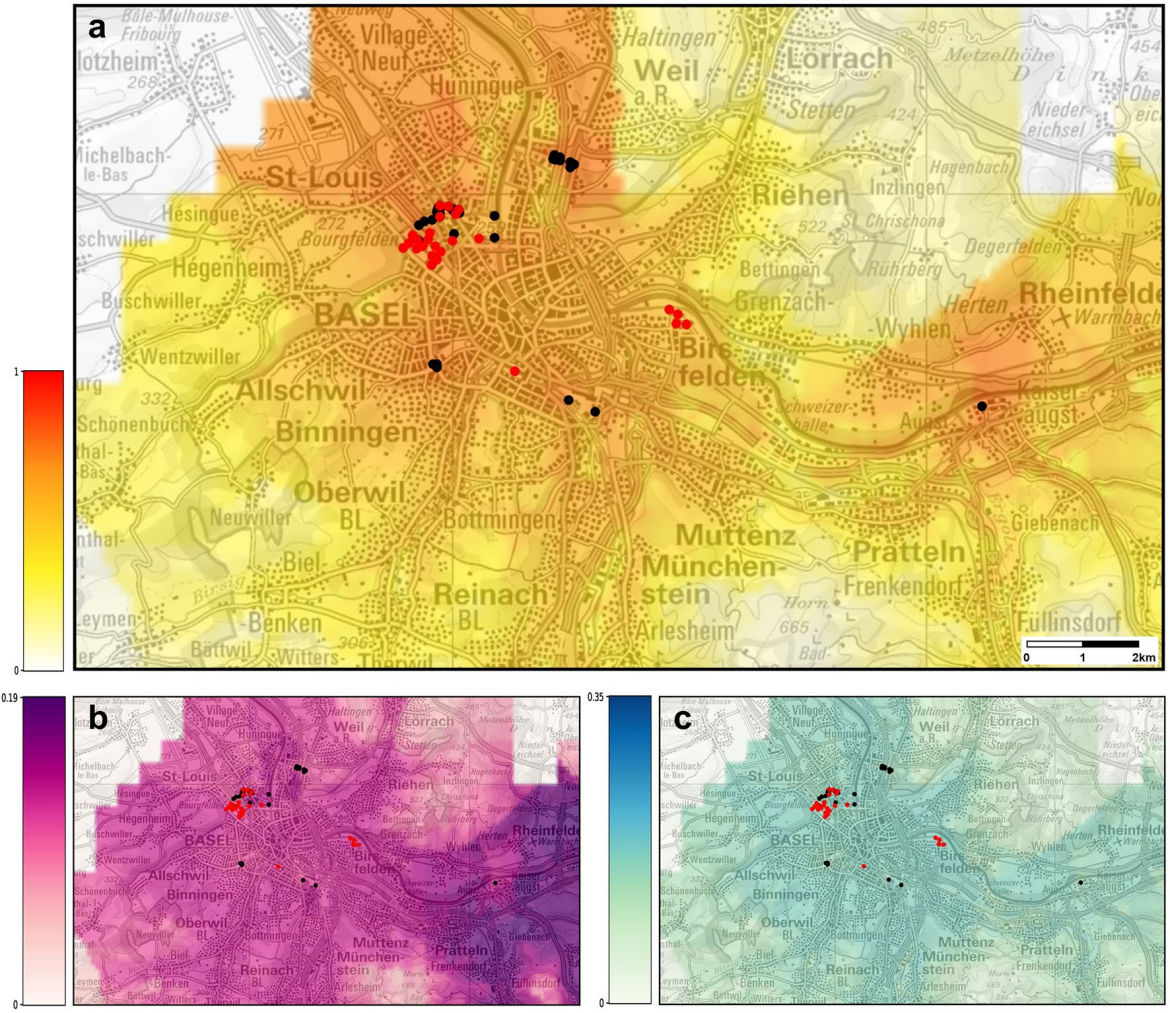
(**a**) Probabilities of establishment of *Ae. albopictus* in Basel using the temperatures corrected with the microclimatic model. The map shows the average risk estimate over the years 2015–2018. The color gradient shows the probability of establishment from 0 (white) to 1 (red). (**b**) Uncertainty of prediction (higher values representing more uncertain predictions). (**c**) The difference between the risk estimates obtained using the original temperatures and the transformed ones. Black and red dots: ovitraps with observation and establishment of *Ae. albopictus* in 2019 and 2021, respectively. Maps modified from https://map.geoadmin.ch/ (accessed on 1 April 2022).

**Figure 6:**
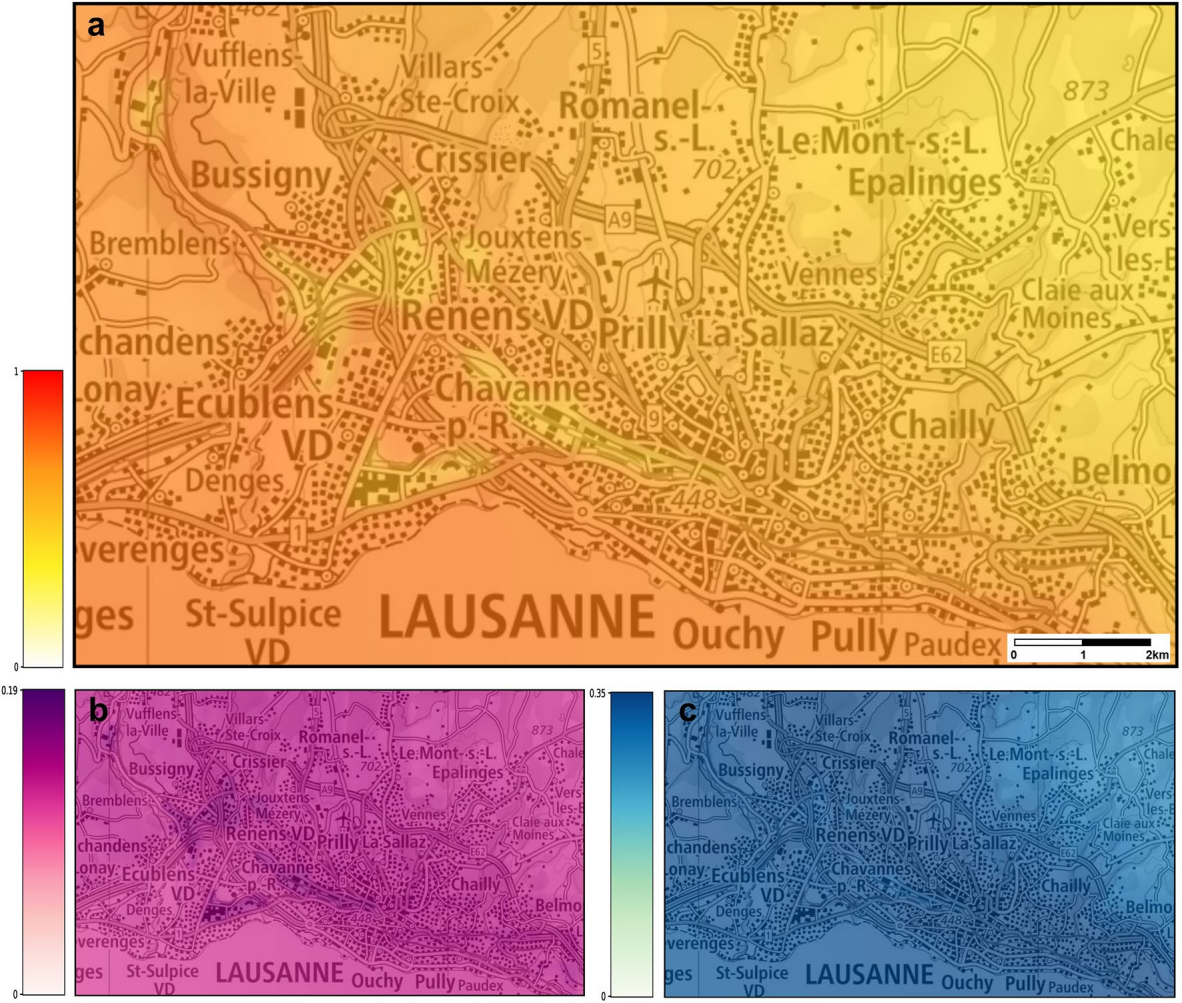
(**a**) Probabilities of establishment of *Ae. albopictus* in Lausanne using the temperatures corrected with the microclimatic model. The map shows the average risk estimate over the years 2015–2018. The color gradient shows the probability of establishment from 0 (white) to 1 (red). (**b**) Uncertainty of prediction (higher values representing more uncertain predictions). (**c**) The difference between the risk estimates obtained using the original temperatures and the transformed ones. Maps modified from https://map.geoadmin.ch/ (accessed on 1 April 2022).

**Figure 7:**
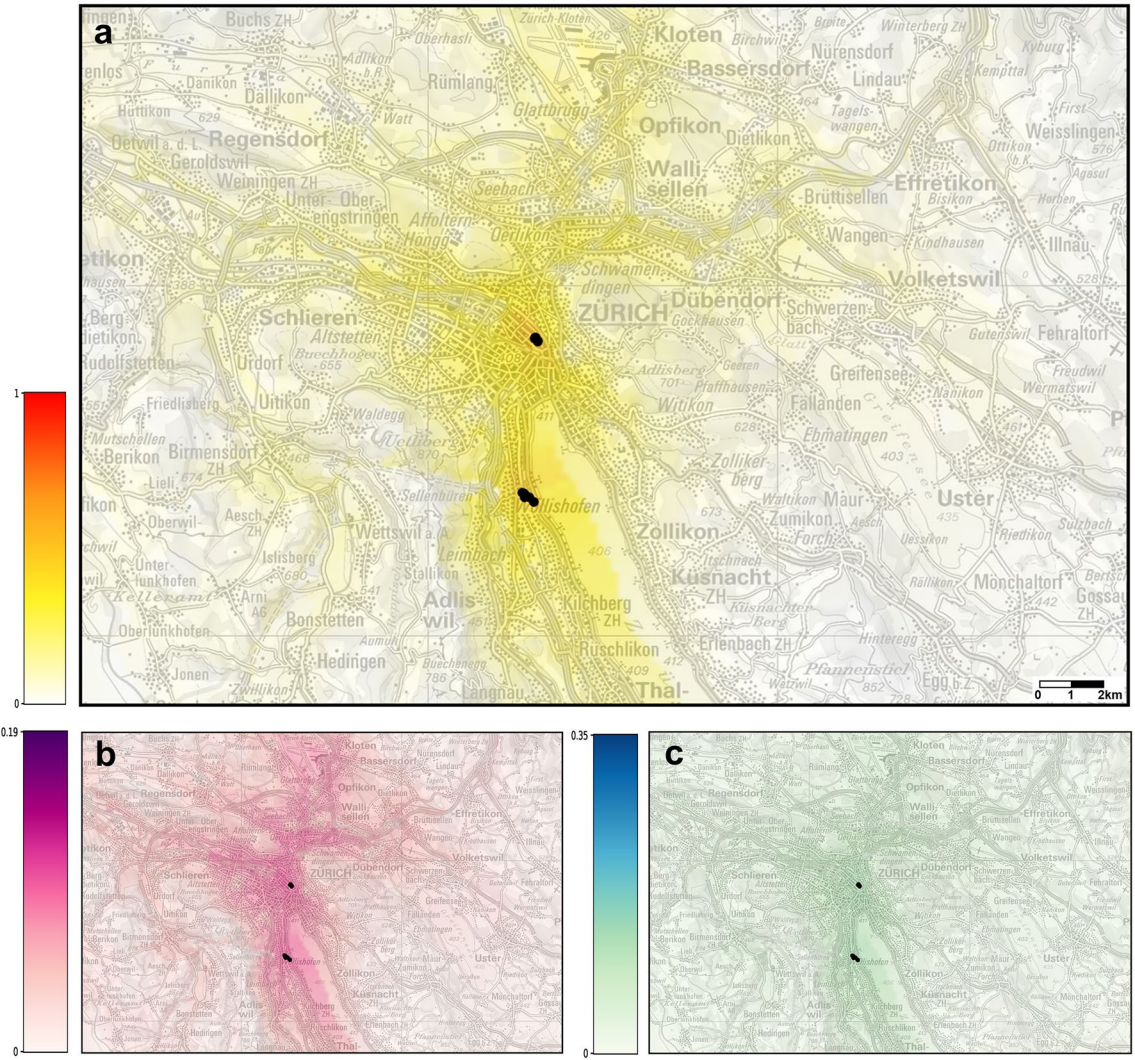
(**a**) Probabilities of establishment of *Ae. albopictus* in Zurich using the temperatures corrected with the microclimatic model. The map shows the average risk estimate over the years 2015–2018. The color gradient shows the probability of establishment from 0 (white) to 1 (red). (**b**) Uncertainty of prediction (higher values representing more uncertain predictions). (**c**) The difference between the risk estimates obtained using the original temperatures and the transformed ones. Black dots: ovitraps with observation of *Ae. albopictus* in 2019. Maps modified from https://map.geoadmin.ch/ (accessed on 1 April 2022).

After incorporating the corrected temperatures based on the microclimatic analysis into the previously developed ecological niche model, we obtained suitability maps for the cities of Basel, Lausanne and Zurich (Figs. 5a, 6a and 7a). The maps show the positions of ovitraps where *Ae. albopictus* was recorded in 2019 (black dots), which were used to compute the feature car distance to establishment (distance from the closest cell with establishment in the previous year). Red dots show the positions where *Ae. albopictus* was established in the most recent surveillance, in 2021 (source of data same as for 2019 data). The red dots are only present in the suitability map for Basel, since no establishment was observed in Lausanne and Zurich in 2021. Each risk map is accompanied by an uncertainty map showing the normalized standard deviation of the predictions used in the corresponding map (Figs. 5b, 6b and 7b). Uncertainty maps describe the reliability of the prediction, higher values of the standard deviation meaning that the prediction is less reliable. The third type of map (Figs. 5c, 6c and 7c) shows the difference between the risk estimates obtained using the original temperatures and the transformed ones. For consistency, the same scale is applied to all maps of comparison between risk estimates and all uncertainty maps.

## Discussion

The aim of this study was to understand if and how the predictions of an ecological niche model based on meteorological climatic data with a relatively large spatial scale (i.e., 1 km^2^) changed when we considered the temperatures of microclimatic habitats. In particular, we wanted to know if we could obtain more precise and realistic risk scenarios for the spread of the mosquito *Ae. albopictus* in temperate urban areas when considering the winter microclimatic conditions of catch basins. Catch basins are considered major sites of oviposition and egg overwintering for *Ae. albopictus* in temperate urban areas^19^. Indeed, the presence of warmer winter climatic conditions in these microhabitats, compared to conditions encountered outside, could favour the survival of diapausing eggs to cold temperatures, promoting in turn the establishment of the mosquito in new areas.

In order to monitor the microclimatic temperatures in catch basins, we developed and implemented a Wireless Sensor Network (WSN) based on the Internet of Things (IoT) approach^29^. This approach is increasingly used for monitoring the environment in various contexts and for various purposes, including vector management. For example, an autonomous WSN was recently developed by Petríc and collaborators^31^ for investigating climatic suitability for the establishment of *Ae. albopictus* in a field site in Belgium. In the present study, the setting up of the WSN was itself a challenge. Indeed, the position of the sensors under the ground surface, inside the catch basins, in addition to the presence of the catch basin’s grid, strongly decreased the signal of the network’s antennas, essential in transmitting the data from the sensor device to our server. This issue has been faced in other studies as well^32^. In the present study, data collected by the sensor devices were also stored on the SD card of the sensor as backup in case of poor data transmission. For our analyses, we used the data stored on the SD cards to work on the complete hourly temperature dataset.

Microclimatic monitoring of catch basins was performed throughout a temperate cold season (i.e., from the beginning of December 2019 to the end of February 2020) in the Swiss cities of Lugano (south of the Alps) and Basel, Lausanne and Zurich (north of the Alps). During this period, temperatures in catch basins, both in urban and peri-urban context, were warmer compared to local meteorological temperatures. The daily temperature fluctuation was always significantly smaller and the daily minimum temperature was almost always significantly higher in catch basins compared to the gridded temperature data, except in three cases. Thus, catch basins in cities seem to provide a thermal buffering during the cold season, allowing warmer temperatures compared to the more exposed and colder temperatures recorded at permanent weather stations. The results confirmed previous reports^19,20,23^ that catch basins of urban stormwater drains can provide favourable conditions for overwintering of diapausing eggs compared to more cold-exposed sites, acting as a sort of microclimate refugia, where the diapausing eggs can persist despite the surroundings being inhospitable.

Previous laboratory experiments on egg mortality in response to cold temperatures showed that minimal survival temperatures for temperate *Ae. albopictus* diapausing eggs were − 10 °C for long-term exposure (i.e., 12–24 h) and − 12 °C for short-term exposure (i.e., 1 h)^33^. This indicated that temperate *Ae. albopictus* diapausing eggs are able to survive winter nights down to −10 °C, although only around 10% of eggs hatched after exposure to this temperature. The percentage of surviving eggs increased to 25% after exposition to − 7 °C for 24 h and to 45% after 24 h at − 5°C^33^. Daily minimum temperatures in the four Swiss cities investigated during the 2019–2020 cold season never dropped below − 5 °C. The coldest temperature measured in catch basins was −3.18 °C and the coldest temperature for the gridded temperature data was −4.67 °C. Indeed, the winter 2019–2020 has been the mildest winter in Switzerland since measurements began in 1864. The national average winter temperature was 0.7 °C, which is almost 3 °C higher than the average of the years 1981–2010 (source: MeteoSwiss). However, most of the Swiss winters in the last decade have been characterized by an average temperature higher than the past. Climate warming is clearly happening in Switzerland. Surface air temperature has increased in all regions of the country since the start of the instrumental record in 1864. Future climate scenarios for Switzerland include increasing mean winter temperatures and decrease in the number of intense cold waves, frost days, and ice days^34^. Therefore, the results obtained for winter 2019–2020 can be considered representative of the most recent years and probably also realistic for the coming decade.

The relation between the cold-season temperatures recorded in catch basins and the gridded temperatures differed among the cities. The catch basins in Basel and Zurich showed a more pronounced mitigating effect over external temperatures changes than the catch basins in Lugano. The mitigating effect of catch basins in Lausanne was halfway between the others. The size of the catch basins could play a role in the difference. The catch basins monitored in Basel and Zurich had a greater depth than the catch basins monitored in Lugano and previous investigations have shown that temperatures measured at 2-m depth inside catch basins were more stable than temperatures measured at 0.3-m depth^23^. However, in the present study the sensors were installed at a depth ranging from 0.3 to 0.5 m, so we don’t know exactly if an effect could be present at this depth. The variation in the mitigating effect of catch basins in different cities could also be linked to the dissimilarity in the cities’ underground infrastructure. Similar to the Urban Heat Island (UHI) effect in the atmosphere of cities, urbanization leads to a warming of the subsurface beneath the cities, a so-called Subsurface Urban Heat Island (SUHI) effect, whose impact is probably area specific^35^. The SUHI effect is caused by heat fluxes from warmed basements, pavements and buried infrastructures such as underground traffic lines as well as industrial and residential subsurface buildings. The effect has been shown both in Basel^36^ and in Zurich^37^ and also in Lausanne (https://geoeg.net/en/2021/07/20/quantification-of-subsurface-urban-heat-islands-and-potential-for-energy-savings-in-lausanne-switzerland/amp/, accessed on 8 April 2022). In Lugano, to our knowledge, it has not been measured but we expect that it is present at a smaller scale compared to the other cities, since the underground structures are less developed. Although we could not find studies on the potential impact of SUHI on the temperature inside catch basins, the varying degrees of the phenomenon in different cities could suggest a different warming impact of SUHI on the temperature of urban catch basins.

The validity of a data-driven model is limited to the area where the data used for training has been collected. When used outside that area, e.g., when used to estimate the risk in regions where mosquitoes have not yet established, the model is extrapolating. Its predictions are therefore valid only under the assumption that the relation between predictors and outcome are the same in the training area as in the area of prediction. In this work, we used a microclimate model based on local measures from a network of sensors to test the validity of this assumption for what concerns the relation between local climate and catch basins temperatures. Having found evidence that the relationship between these two sets of variables is different between Ticino (where training data were collected) and other cities, due to a larger mitigating effect on external winter temperatures variation of catch basins in bigger cities, we developed a simple strategy for including this phenomenon in the model, thus correcting the risk predictions issued by the original model. Far from producing accurate estimates of the microclimatic conditions, we believe that this approach gives a more realistic view of the situation as it takes into account, albeit in an approximate way, the evident differences between the geographical areas considered. If we want to apply the same method to a new city, it is necessary to deploy a specific WSN and, in case the average effect of catch basins on external temperature in these cities is found different than in Ticino, fit the model relating catch basins’ temperatures to gridded temperatures to the data collected for that city to find the parameters ${a}_{c}$ and ${b}_{c}$ to be used in Eq. (1).

For all the cities investigated, the risk of establishment of *Ae. albopictus* was higher when the cold-season microclimatic conditions of the catch basins were taken into account into the ecological niche modelling. The importance of the microclimatic conditions seemed to vary between the cities, with the highest impact in Lausanne, followed by Basel, while the impact did not seem to be very high in Zurich (Figs. 5c, 6c and 7c). Due to the peculiar shape of the logistic function used by the ensemble model to transform the (linear) log-odds to a risk measure, the effect of the temperature’s transformation is reduced when the risk is either very low (i.e., close to zero) or very high (i.e., close to one). This explains why in Zurich, the city with the smallest estimated risk among the three, the effect of the correction is small. On the other hand, in Lausanne the risk is always in an intermediate range (between 0.1 and 0.5) since, in the lack of establishment measures, we have assumed that all cells are close to an established cell (i.e., at a travel distance by car of 30 s). This is one reason why, the change induced by the correction in the risk predictions for Lausanne is large everywhere. It should be recalled also that, among the predictors used in the ecological niche model, the 25^th^ percentile of the maximum daily temperatures during the cold season corresponds to the second largest coefficient (see Fig. 2 of^28^). Therefore, the correction for catch basins microclimates applied to the maximum daily temperature series is the one that affects the most the predictions of the model. In Fig. 5, one can see that the largest correction of the maximum daily temperature is for Lausanne, whereas the smallest is for Zurich, which further explains the different increase in the estimated risk for these two cities.

The risk of establishment is also high in the urban areas of Basel, while in Zurich the establishment of *Ae. albopictus* seems less probable. At the model level, the main effect of the difference between the risk in Basel and Zurich is due to the car distances considered, which were greater in Zurich. Another effect on the difference is due to the fact that the winter maximum temperatures and summer rainfall are lower in Zurich for the years 2015–2018 to which the model was applied. From a practical point of view, the difference between the risk in Basel and Zurich could be explained also by the geographical position of the cities and the presence of the mosquito itself. Basel is adjacent to existing *Ae. albopictus* colonies, both on the French and German border with Switzerland^38^, which means that the area is constantly under pressure of introduction of mosquitoes from these external populations^39^. This situation is very similar to what happens in Ticino with the constant introduction pressure from nearby Italy, where *Ae. albopictus* is not actively controlled^26^. Differently, in Zurich this introduction pressure from close external populations is much lower at present (the same can be said for Lausanne). Indeed, the small population detected in 2018 in a suburban neighbourhood in the Wollishofen district was geographically isolated and could be well managed by immediate surveillance and control efforts^40^.

This study has limitations. First, risk predictions for Basel, Lausanne and Zurich are extrapolations based on a model developed from data from Ticino. They are made, therefore, under the assumption that the relation between predictor variable and mosquitoes’ establishment observed in Ticino can be generalized to the other cities. Also, while establishment data have been collected across all Ticino, only Lugano catch basins have been considered for modeling microhabitat conditions. Furthermore, a limited number of catch basins has been considered for each city, and their choice was not completely random, but constrained to their accessibility and presence of a good quality of the network signal inside them, limiting the robustness of the analyses. However, the catch basins were sampled across different and varied areas of the studied regions and so they should give an acceptable estimate of the average catch basin’s microclimatic behavior in a city. Therefore, despite the effort made to select representative catch basins, the model developed for the catch basins temperatures can only represent a rough approximation of the real microhabitat conditions. Finally, the linear correction implemented to account for local temperatures cannot capture all the actual implications of microhabitats. Despite all these limitations, the results showed that the risk of establishment of *Ae. albopictus* did increase, in the three urban areas of interest, when we considered the winter temperatures of catch basins, which are typical urban diapausing microhabitats. The use of an average model of the temperatures across multiple microhabitats (i.e., different catch basins of the same city in the present study) should provide a more accurate representation of the winter conditions faced by diapausing eggs than the meteorological station data or spatial climates interpolated from the meteorological station data. The results provide some indications which are worth considering when developing *Ae. albopictus* monitoring and containment strategies.

## Conclusions

Most ecological niche models used to predict the distribution of invasive mosquitoes are based on climate data originating from weather stations or remotely sensed land surface temperature data. The present study shows that cold-season temperatures recorded in potential overwintering microhabitats (i.e., catch basins) of *Ae. albopictus* eggs in temperate cities are warmer and therefore more favorable for the survival of eggs than temperatures recorded at the local level by weather stations. This indicates that the local climate data might not mirror the microclimatic conditions experienced by the organisms within their habitats. Comparison of the predictive model’s results based on local climate data and microclimate data indicate that the risk of establishment for *Ae. albopictus* in temperate urban areas increases when microhabitat temperatures are taken into account. Using standard meteorological temperatures in temperate climates might lead to an underestimation of the risk of establishment of this vector in urban areas. In each city examined, we observed a linear relationship between catch basins’ temperatures and gridded temperatures. This allowed us to fit a mixed effects model and use it to transform the gridded temperatures so to mimic the average behaviors of the observed catch basins. Since the relation between catch basin’s microclimates and gridded temperatures differed significantly among the cities, the prediction of risk establishment in other cities will require the installation of a WSN in order to find out the appropriate parameters to transform the local temperature data. The predictive maps of climate suitability obtained in the present study can be used by the national multidisciplinary network for the control of invasive mosquitoes in Switzerland (Swiss Mosquito Network, http://www.mosquitoes-switzerland.ch (accessed on 12 April 2022)) to aid in prioritizing mosquito surveillance and control at the local level.

## Materials and methods

### Study areas

The study took place in the cities of Basel, Lausanne, Lugano and Zurich, in Switzerland. Basel, Lausanne and Zurich are located north of the Alps, in the geographical region of the Central Plateau (Supplementary Fig. S1). This region stretches from Lake Geneva in the southwest to Lake Constance in the northeast and is the most densely populated region in Switzerland. Zurich is the largest city of Switzerland and encompasses 88 km^2^ with a total human resident population of 420,217^41^. Lausanne and Basel are smaller than Zurich, with a surface of 41 and 24 km^2^ and a total population of 139,408 and 173,232, respectively^41^. The climate in these three cities is moderately continental, with cold winters often reaching freezing temperatures in January, and warm summers. Lugano is located in Ticino, south of the Alps (Supplementary Fig. S1), where the climate is strongly affected by the Mediterranean Sea, with mild winters and summers warm and humid, sometimes hot. Lugano is the smallest of the four cities with 50,603 residents in 26 km^2^^41^.

*Aedes albopictus* is well established in Lugano since 2009 and an integrated vector management is constantly implemented to contain the numbers of the mosquito at a manageable level. This consists of an intensive surveillance, with oviposition traps distributed according to a grid system, several control interventions, such as the removal of breeding sites and the systematic application of larvicides in public areas, mainly in catch basins, and extensive public information campaigns^24,26^. In Basel, two populations of *Ae. albopictus* are established since 2018: a first population in an area adjacent to the motorway toll on the border with France and a second population in an area near the border with Germany^27^. The mosquito has also been recorded repeatedly at various locations in the city of Basel and the surveillance indicates that the mosquito is spreading^42^. Control actions are taken exclusively within the perimeter of repeated detections of the mosquito and include regular treatment of catch basins with larvicides, distribution of flyers and door-to-door information campaigns^42^. In Zurich, *Ae. albopictus* was first detected in 2016 in a bus station for international coach services located in the centre of the city, near the main train station. Thanks to immediate surveillance and control actions (i.e., treatment of catch basins in the area with larvicides), to date there is no established population within the perimeter of the bus station despite continuous repeated introductions^40^. A small population was also detected in 2018 in a suburban neighbourhood in the Wollishofen district of Zurich, approximately 5 km southwest from the international bus station. Also in this case, immediate surveillance and control actions, including larval control and door-to-door information, were taken with success and no adults, eggs or aquatic stages have been found in 2020 and 2021^40^. In Lausanne, no tiger mosquito has been reported to date (Swiss Mosquito Network, http://www.mosquitoes-switzerland.ch (accessed on 17 February 2022)).

### Microclimate data

Based on a previous investigation we conducted in Ticino, Basel and Zurich^20^, we focused the microclimate monitoring on ordinary stormwater catch basins positioned on the side of public roads. In each city, we monitored ten catch basins located either in urban context (defined as areas with high-density development, consisting of apartment blocks, commercial or industrial units) or in residential areas consisting mainly of houses with private gardens located in peri-urban area (Supplementary Table S1, Supplementary Fig. S2). The catch basins were usually homogeneous in dimension, in the same city, although we recorded variations in depth. In Basel, we included catch basins located in the urban area near the border with France, in which *Ae. albopictus* is established. In Zurich, we included catch basins located in the international bus station, where *Ae. albopictus* was recorded in summer, and in the residential area of Wollishofen, where a small population of *Ae. albopictus* was detected and then likely eradicated. In Lausanne, some catch basins were selected in potential points of introduction of the mosquito (e.g., near a campsite, the main train station, etc.). In Lugano, *Ae. albopictus* was established in all the locations selected.

A sensor device was installed in each selected catch basin. The sensor devices were built in house. The development of the devices and the Wireless Sensor Network (WSN) has been described in detail by Strigaro et al.^29^. Briefly, the device consisted of a waterproof plastic box containing a LoPy Micro-Controller Unit (Pycom, Guildford, United Kingdom), a waterproof temperature probe (accuracy of ± 0.5 °C), a light sensor (measuring illuminance arriving at the sensor device, in lux), an SD card, the rechargeable batteries and other parts. The main box, with the light sensor, was hung on the inside wall of the catch basin. The temperature probe was attached to the wall at a depth ranging from 0.3 to 0.5 m, depending on the depth of the catch basin and the level of the water in the catch basin. The probe was placed in direct contact with the inside wall of the catch basin, in order to measure the microclimatic conditions where the mosquito eggs are potentially laid. The data collected was transmitted to a data warehouse based on istSOS, an open-source Python based implementation of the Sensor Observation Service standard (SOS) of the Open Geospatial Consortium (OGC)^43^. The data was transmitted through the Swisscom Low Power Network (LPN) LoRaWAN (Swisscom Ltd, Ittigen, Switzerland): the data sent by the sensor devices was received by a Swisscom Gateway and then sent to the data warehouse^29^.

In addition to the sensor devices installed in the catch basins, four devices were installed outside four catch basins in each city, except in Lugano, where three devices were installed. These external devices were placed in vegetation representing potential resting habitats for *Ae. albopictus* adults in the reproductive season*,* at 1–2 m above the ground and analyzed to confirm the close similarity between measured external temperatures and MeteoSwiss gridded temperature data. However, since the main goal of the data collection was to model the differences between MeteoSwiss gridded temperature data and catch basins’ temperatures, only a small number of external sensors were deployed. Microclimate data were collected from beginning of December 2019 to end of February 2020, a period defined as cold season, with acquisition interval set at one hour. In Lugano, data collection started on the 12th or 13th of December 2019.

### Local climate data

We used two types of local climate data. The first type is the momentary hourly free-air temperatures recorded at 2 m above ground level by permanent weather stations. The weather stations belong to SwissMetNet, the automatic monitoring network of MeteoSwiss. For each city, we selected the weather station closest to the study area (Supplementary Table S1, Supplementary Fig. S2) and temperature data were retrieved from https://gate.meteoswiss.ch/idaweb (source: MeteoSwiss, Zurich-Airport, Switzerland; accessed on 12 August 2021).

The second type of local climate data is the MeteoSwiss spatial climate daily datasets (source: MeteoSwiss). These temperature datasets are constructed through interpolation of daily minimum, maximum, and mean temperatures from a network of approximately 90 SwissMetNet permanent weather stations to a 1 km resolution grid in the Swiss coordinate system CH1903^44,45^. This results in three temperature datasets describing the km-scale distribution of day-to-day temperature variations in Switzerland. We referred to them as gridded temperature data. Each monitored catch basin and external device was assigned, based on its geographical position, to the corresponding 1 km × 1 km cell of the climate grid. Each cell was identified with its MeteoSwiss (MS) number (Supplementary Table S1).

### Data analysis

The hourly temperatures were used to compute daily mean, maximum and minimum temperatures and daily temperature ranges, which were calculated as the difference between the maximum and minimum daily temperature. Temperatures of catch basins and external habitats were compared to temperatures of permanent weather stations and to the gridded temperatures both graphically and using the nonparametric Mann–Whitney U-test, for which a *P* value of < 0.05 was determined as significant. For these calculations and comparisons, we used the softwares Microsoft Excel (Microsoft Corporation, Redmond, USA) and IBM SPSS Statistics for Windows, Version 26.0 (IBM Corporation, Armonk, USA).

The relation between catch basins and gridded temperatures was further investigated, using scatterplots, for each data series (i.e., daily average, minimum and maximum temperatures). The association between each temperature series obtained from the sensors of the WSN and the corresponding gridded temperature series was analyzed independently for each catch basin using a simple linear regression model:

$$ T_{i} = m_{i} T_{MS} + q_{i} , $$

where ${T}_{i}$ is the temperature of the i-th catch basin, ${T}_{MS}$ the gridded temperature of the corresponding cell and ${m}_{i}$ and ${q}_{i}$, respectively, the slope and intercept of the linear model. For comparison, similar models were built also for the relation between gridded temperatures and weather stations and external sensors temperature measurements. The coefficients (slope and intercept) of the individual catch basin microclimate models were then plotted on a scatterplot to compare their distribution across cities. The differences between these coefficients in the different cities were also jointly analyzed using a MANOVA hypothesis test.

After the independent analysis of each individual catch basin, we built, for each temperature series, a global model of the relation between gridded temperatures (predictor) and catch basin temperatures (response). To account for the difference between catch basins and between cities, we considered a hierarchical model grouping data based on the city (first level) and the catch basin (second level). We assume random effects intercept and slope both for the city and the catch basin predictors. Let *y*_*c,m*_ be the daily temperature (either minimum, maximum or average) observed in catch basin *m* of city *c*, and *x* the temperature from the corresponding cell of the gridded temperature model. The resulting mixed effect model can be written as follows

$$ y_{c,m} = a_{0} + b_{0} x + a_{c} + b_{c} x + a_{m} + b_{m} x , $$

where ${a}_{0}$ and ${b}_{0}$ are the fixed effects, ${a}_{c}$ and ${b}_{c}$ the random effects associated to the city $c$ and ${a}_{m}$ and ${b}_{m}$ the random effects associated to the individual catch basing $m$. To further confirm the presence of a significant effect of cities on catch basins temperatures, we computed the normalized relative likelihood^30^ of the mixed effects model including both the city and catch basin random effects versus the mixed effects model including only catch basin random effects.

To describe the average relation between microclimatic and gridded temperatures in each city, we dropped the terms associated to the catch basin random effects, i.e., ${a}_{m}$ and ${b}_{m}x$, and produced from the available gridded temperatures, estimates of the average catch basin temperatures in each cell of city $c$ as follows:

1 $$ y_{c} = a_{0} + b_{0} x + a_{c} + b_{c} x. $$

The corrected temperatures were incorporated into the previously developed ecological niche model, which has been described in detail in Ravasi et al.^28^. Briefly, the ecological niche model consists in an ensemble of Lasso-regularized logistic regression models that use the long-term dataset of *Ae. albopictus* presence–absence records in Ticino from 2005 to 2012 as response variable in the training process. The model evaluated 79 explanatory variables, which included indicators of terrain morphology, land use coverage, total human population, meteorological temperatures and precipitations and travel distance (by car) from the nearest 200 m × 200 m cell with *Ae. albopictus* establishment. The performance of the model in predicting the probability of establishment of *Ae. albopictus* in Ticino was assessed both by cross validation over the training years 2007 to 2012 and on the test years 2013, 2014, and 2015. On the year 2007 to 2012, which represent the period of diffusion of *Ae. albopictus* in Ticino, the model obtained an AUC above 0.85, whereas on years 2013 to 2015, which refers to a period were mosquitoes were well established (less interesting, therefore, for the purpose of predicting establishment), the AUC was almost 0.75. The ensemble model was then used to extrapolate the probability of establishment in the cities of Basel and Zurich starting from the initial observations of mosquitoes’ establishment collected in 2019, and using meteorological data from the years 2015, 2016, 2017 and 2018 as scenarios for the meteorological conditions in the coming years^28^. For each of the four scenarios and each of the eight models of the ensemble, a risk prediction was produced for each 200 × 200 m cell of the areas of interest. Finally, a single risk prediction for each cell and a measure of its uncertainty were obtained, respectively, as the average and the standard deviation of these 32 risk estimates and projected onto a suitability map showing the average risk estimates and an uncertainty map showing the standard deviations.

Ten explanatory features were identified as most informative for the prediction of establishment of *Ae. albopictus* in Ticino^28^, by at least half of the eight ensemble models; among them, two were related to cold-season temperatures. It appeared therefore important to account for the results obtained from the microclimate analysis when predicting the risk of the establishment. To incorporate these results, gridded temperatures relative to the cold season should have been transformed based on the model in Eq. (1) before deriving the cold-season temperature predictors used in Ravasi et al.^28^. However, since these predictors are linear functions of the original temperature series, the linear transformation in Eq. (1) can be applied directly to them. The natural approach to account for cold-season catch basin temperatures would then be the following: (i) transform the predictors related to the cold-season temperatures in the training dataset from Ticino using Eq. (1) with the random effect coefficients ${a}_{c}$ and ${b}_{c}$ estimated for *c* = Lugano; (ii) re-train the individual models of the environmental niche ensemble model using the transformed training dataset; (iii) transform the cold-season temperature related predictors for Basel, Lausanne and Zurich of the years 2015, 2016, 2017 and 2018 (i.e., the reference years used to develop the risk maps) using Eq. (1) with the random effect coefficients ${a}_{c}$ and ${b}_{c}$ estimated for *c* = Basel, Lausanne, Zurich; (iv) apply the model trained in (ii) to the dataset including the transformed cold-season temperature predictors for Basel, Lausanne, Zurich to obtain corrected risk predictions of *Ae. albopictus* establishment in those cities. However, since in Ravasi et al.^28^ the original features were rescaled in the range [0, 1] (minmax scaling) before learning the individual models of the ensemble, steps (i) and (ii) would not affect their parameters nor change the set of predictors selected but would only result in different values of the minmax scaler parameters related to the cold-season temperature predictors. Therefore, we simplified the above procedure by skipping steps (i) and (ii) and modifying step (iii) as follows. First, the cold-season temperature related predictors for the cities of Basel, Lausanne or Zurich are converted into average catch basin temperatures using Eq. (1) with the random effect coefficients ${a}_{c}$ and ${b}_{c}$ of each city; then, the average catch basin temperatures estimated for Basel, Lausanne and Zurich are inversely transformed according to Eq. (1) using, this time, the random effect coefficients ${a}_{c}$ and ${b}_{c}$ found for Lugano. Such transformed predictors should be interpreted as the gridded cold-season temperatures that, in Lugano, would produce (estimated) catch basins condition equal to those obtained for Basel, Lausanne or Zurich in correspondence of the gridded cold-season temperatures considered for these cities (i.e., those observed in the years 2015, 2016, 2017 and 2018).

For Basel and Zurich, the feature “car distance to establishment” was computed using the records of positive oviposition traps for 2019 (37 points of first establishment for Basel and 16 for Zurich; data kindly provided by the Inspection body for chemical and biosafety (KCB) of the Cantonal Laboratory of Basel-Stadt, the Swiss Tropical and Public Health Institute, the Urban Pest Advisory Service of the City of Zurich, and the Office for Waste, Water, Energy and Air (AWEL) of the Canton of Zurich). The feature was built by computing the smallest distance between each cell of Basel or Zurich and the identified position of 2019 establishments. In Lausanne, *Ae. albopictus* has not been observed yet. Therefore, it was not possible to use positive oviposition traps to compute the car distance to establishment feature. Coherently with what was done in Ravasi et al.^28^, we kept a fixed car distance to establishment of 0.5 min (30 s) for all cells, implying that establishment has already occurred in the neighborhood. This provided an overview of the areas in Lausanne which are mostly at risk in case a first colony of mosquitoes arrives.

Four types of maps were produced for each city, by projecting the results onto Swiss national maps (Federal Office of Topography swisstopo): (1) a suitability map estimated from the original temperatures (not shown here); (2) a suitability map based on the transformed temperatures; (3) a map showing the difference between risk estimates in (1) and (2); (4) an uncertainty map for the predictions obtained using the transformed temperatures, providing an indication about the reliability of the risk estimates displayed in the suitability map.

Scatterplots, risk maps, MANOVA tests, and regression models were created in Python (version 3.8.12, Python Software Foundation, Wilmington, United States) using packages *matplotlib*, *statsmodels*.

## Supplementary Information

Supplementary Information 1.

Supplementary Information 2.

Supplementary Information 3.

## Data Availability

The microclimate temperature data recorded and analyzed during this study are included in this published article (and its Supplementary Information files). Data used in the ecological niche model was obtained from the different sources cited in our previous publication^28^ and is available with the permission of these sources.
