# Supplementary material for: The effects of microclimatic winter conditions in urban areas on the risk of establishment for Aedes albopictus
Source: Sci Rep. 2022 Sep 24;12:15967. doi: 10.1038/s41598-022-20436-9 (PMC9509395; doi:10.1038/s41598-022-20436-9)
Supplement: Supplementary file 1 — Supplementary Information 1. [file 41598_2022_20436_MOESM1_ESM.pdf]

Supplementary Information

The effects of microclimatic winter conditions in urban areas on the risk of establishment for *Aedes albopictus*

Damiana Ravasi, Francesca Mangili, David Huber, Massimiliano Cannata, Daniele Strigaro, Eleonora Flacio

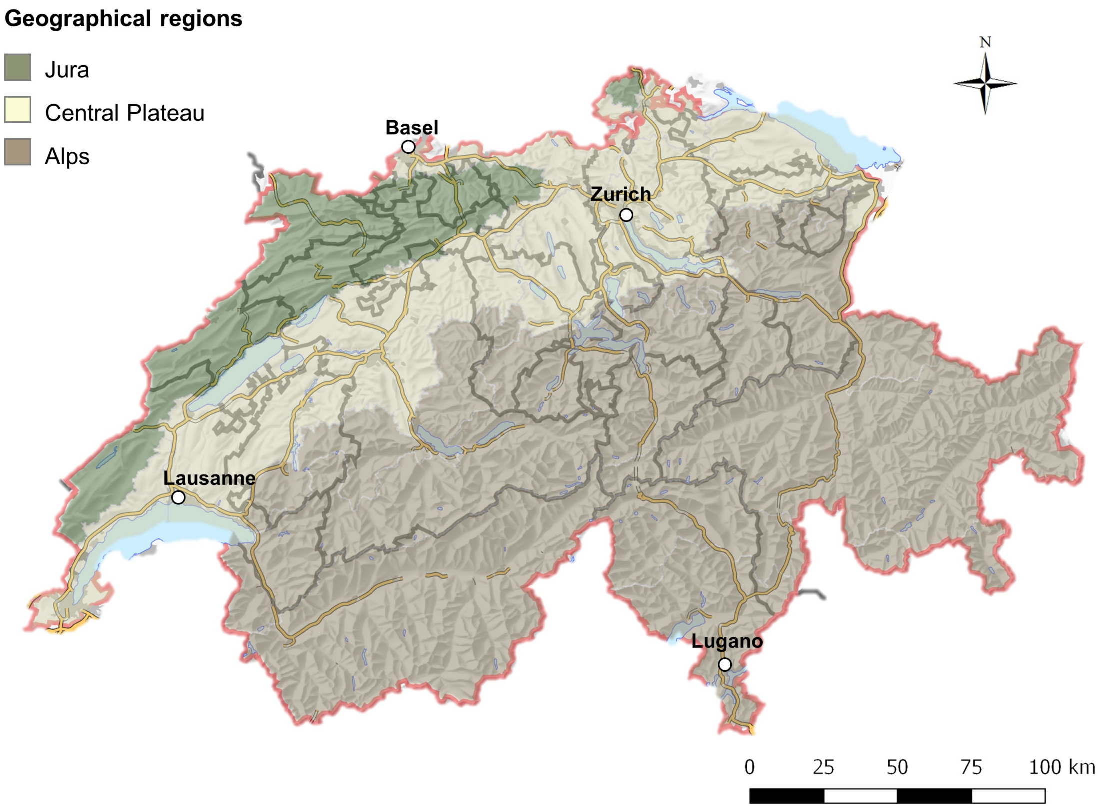

Figure S1. Map of Switzerland showing the three main geographical regions and the four cities studied. Source of main map: Swiss Federal Office of Topography, modified in qGIS 3.0.3. Map of geographical regions by Romano 1246, distributed under a GNU Free Documentation License.

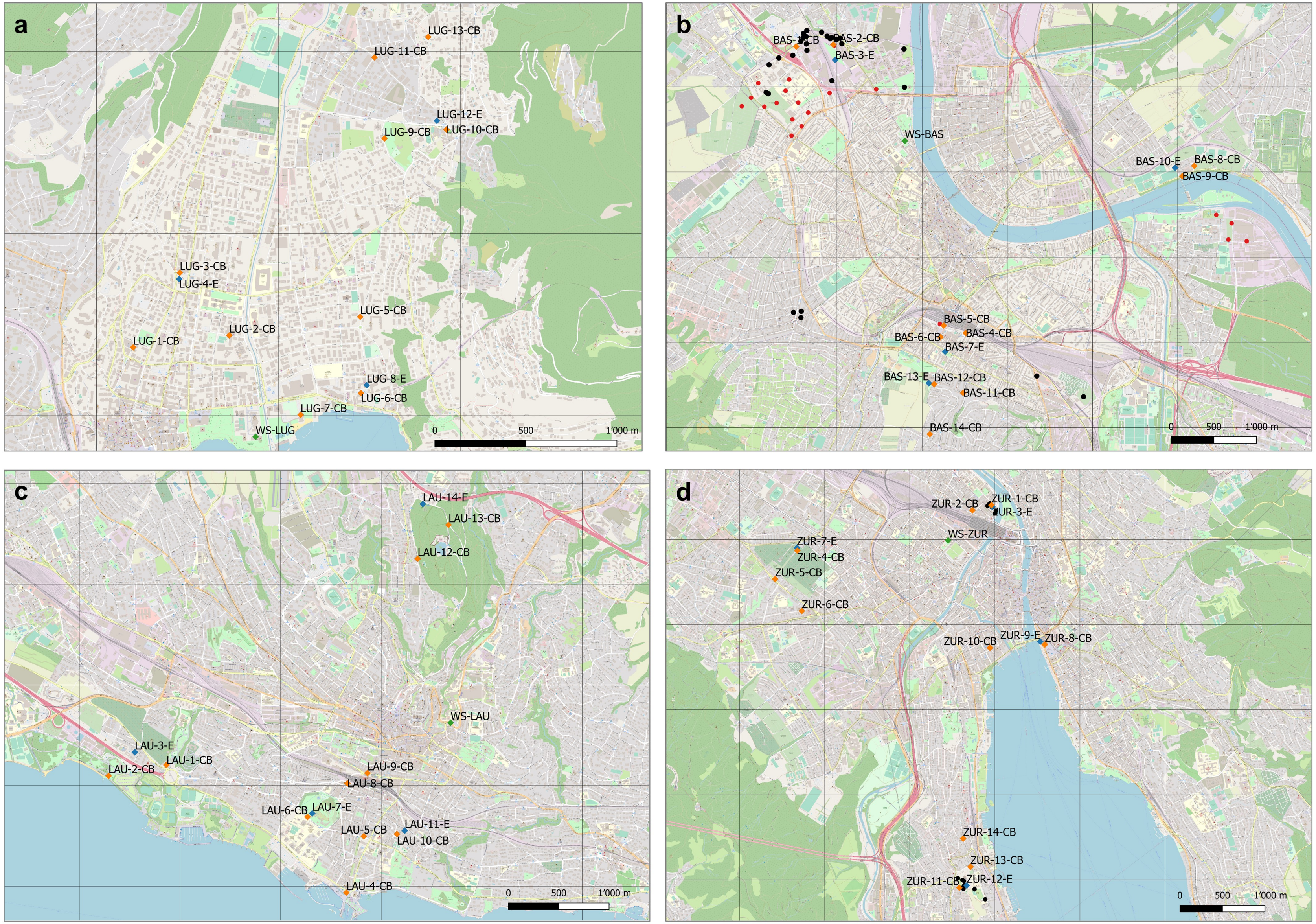

Figure S2. Maps of a) Lugano, b) Basel, c) Lausanne and d) Zurich) with the locations of the weather stations (green diamonds), catch basins (orange diamonds) and external sites (blue diamonds). Black dots in Basel and Zurich represent ovitraps in which Ae. albopictus was observed in 2019. Red dots in Basel represent ovitraps with *Ae. albopictus* establishment in 2021. Source of main map: OpenStreetMap (© OpenStreetMap contributors), modified in qGIS 3.0.3.

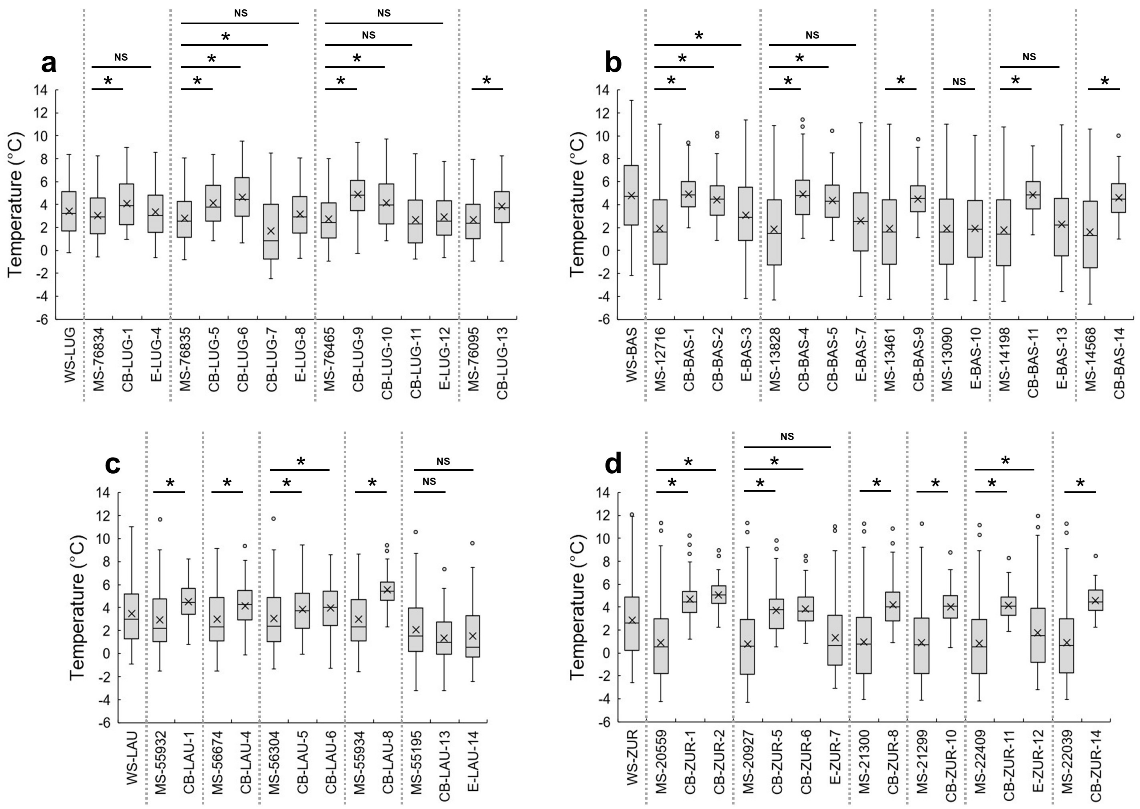

Figure S3. Daily minimum temperatures in the cities of Lugano (a), Basel (b), Lausanne (c) and Zurich (d). The x marks within the boxes represent the means. Temperatures were recorded in permanent weather stations (WS), MeteoSwiss cells (MS) and corresponding catch basins (CB) and external potential warm-season resting habitats (E) (Supplementary Table S1).
*: Significant differences. NS: differences not significant.

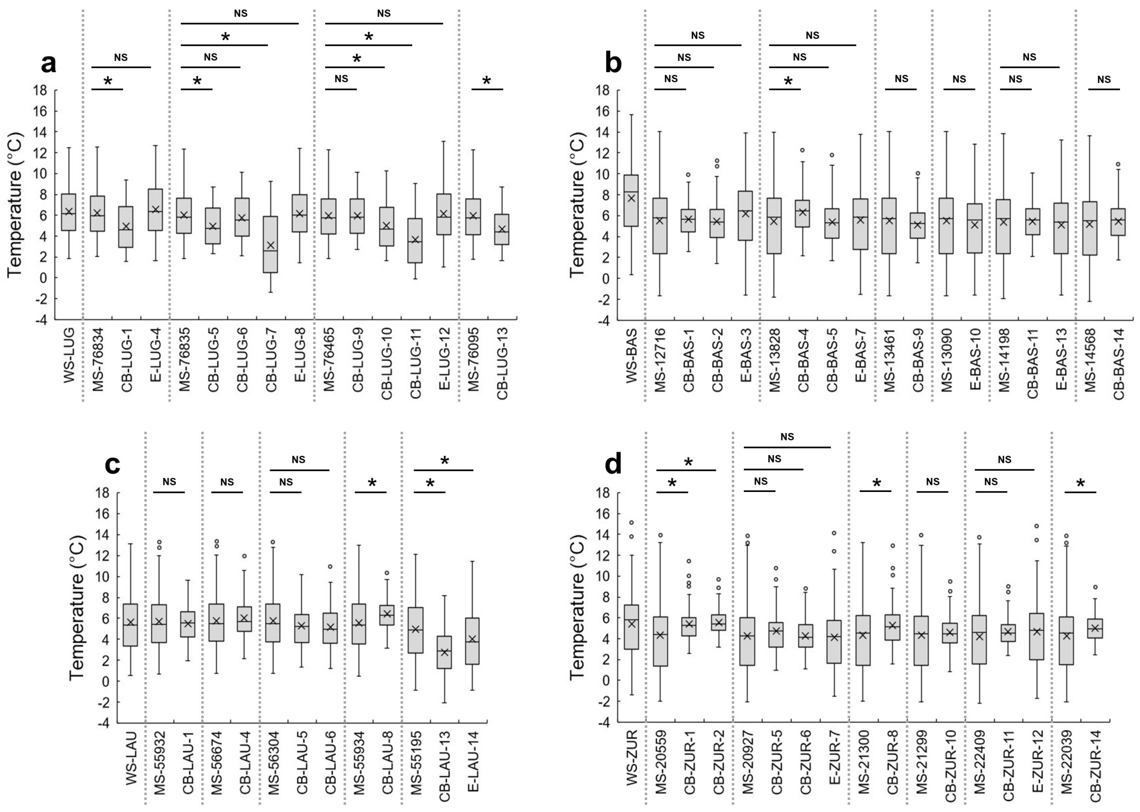

Figure S4. Daily mean temperatures in the cities of Lugano (a), Basel (b), Lausanne (c) and Zurich (d). The x marks within the boxes represent the means. Temperatures were recorded in permanent weather stations (WS), MeteoSwiss cells (MS) and corresponding catch basins (CB) and external potential warm-season resting habitats (E) (Supplementary Table S1).
*: Significant differences. NS: differences not significant.
